# Overexpression of RAB31 in gastric cancer is associated with released exosomes and increased tumor cell invasion and metastasis

**DOI:** 10.1002/cam4.6007

**Published:** 2023-05-24

**Authors:** Shan Wu, Chaotao Tang, Qing‐wei Zhang, Qian Zhuang, Xin Ye, Jie Xia, Yan Shi, Min Ning, Zhi‐xia Dong, Xin‐jian Wan

**Affiliations:** ^1^ Department of Endoscopy Shanghai Jiao Tong University Affiliated Sixth People's Hospital Shanghai China; ^2^ Department of Gastroenterology The First Affiliated Hospital of Nanchang University Nanchang Jiangxi China; ^3^ Division of Gastroenterology and Hepatology, Renji Hospital, Shanghai Institute of Digestive Disease, School of Medicine Shanghai Jiao Tong University Shanghai China

**Keywords:** exosome, gastric cancer, metastasis, PSMA1, RAB31

## Abstract

**Background:**

Gastric cancer (GC) is one of most common cancers worldwide. Several studies have suggested that Rab31 functions as a membrane vesicle transport regulator; however, the mechanism by which RAB31 regulates exosome secretion and promotes metastasis remains to be clarified.

**Methods:**

We examined the expression of RAB31 protein and mRNA in GC tissue samples via immunohistochemistry and reverse transcription‐polymerase chain reaction assays, respectively. We elucidated the function of RAB31 in GC cells by constructing a cell model and a pulmonary metastatic model of GC with overexpression of RAB31. Protein mass spectrometry was used to identify the exosomal protein.

**Results:**

RAB31 expression increased at both the protein and mRNA levels with the development of GC. Cells overexpressing RAB31 showed an enhanced ability to migrate in both the in vitro cell model and the pulmonary metastatic model of GC. Exosome nanoparticle tracking analysis and electron microscopy revealed that the both the number and size of the exosomes secreted by GC cells were reduced when RAB31 expression was depleted. Injection of exosomes derived from RAB31 overexpressing cells promoted pulmonary metastasis in vivo. Analysis of the exosomal proteins revealed that PSMA1 was overexpressed in GC tissue in accordance with RAB31 expression. PSMA1 overexpression was highly associated with poor prognosis of GC patients.

**Conclusion:**

Our findings revealed a key role for RAB31 in GC metastasis through regulation of exosome secretion.

## 1 INTRODUCTION

Gastric cancer (GC) is among the most common and deadliest malignancies, comprising 8.2% of global cancer‐associated mortality.^1^ GC tumors tend to be highly invasive and exhibit high rates of metastatic progression, contributing to poor patient outcomes, although the specific underlying molecular drivers of these outcomes remain to be fully clarified. Additional mechanistic research focused on the factors that govern GC tumor invasivity and metastasis is thus warranted in an effort to guide new approaches to diagnosing and treating this form of cancer.

RAB31 is a monomeric GTP‐binding Ras‐related protein in brain (RAB) family member that we have previously shown to be overexpressed in GC tumor tissues.^2^ RAB31 and associated proteins are also key regulators of membrane vesicle transport activity.^3^, ^4^ We therefore hypothesized that RAB31 may function as a regulator of GC tumor invasivity and metastasis through its ability to control the release of exosomes from these tumor cells.

Exosomes are small membrane‐enclosed vesicles that are released from cells upon intracellular multivesicular body (MVB) fusion with the plasma membrane. Exosomes contain a rich range of macromolecular cargos including nucleic acids and proteins, enabling them to function as a bridge linking cells and their surrounding microenvironmental context. GC tumor‐derived exosomes have previously been suggested to drive enhanced GC metastatic progression through the MAPK pathway, and they have been found to be significantly related to poor prognostic outcomes.^5^

The present study was developed to explore the role that RAB31 plays as a regulator of GC tumor invasivity and metastatic progression through the use of clinical samples and a murine model of GC pulmonary metastasis. Together, these experiments revealed that the overexpression of RAB31 serves as an important mediator of GC tumor invasion and metastasis through its ability to regulate exosome secretion.

## 2 MATERIALS AND METHODS

### 2.1 Clinical patients

In total, 84 GC tumor and healthy paracancerous tissue pairs isolated from individuals that underwent treatment at the Shanghai Sixth People's Hospital (China) from May to November 2015 were used to prepare tissue microarrays. Patients had not undergone radiotherapeutic or chemotherapeutic treatment prior to tissue collection. All patients underwent telephone‐based follow‐up for 1–75 months. Endoscopy procedures were additionally used to collect 41 paired GC tumor and paracancerous tissue samples for qPCR analyses. The Ethics committee of Shanghai Sixth People's Hospital affiliated with the School of Medicine of Shanghai Jiao Tong University approved this study, and all participants provided written informed consent to participate. For further verification, the clinical data information were obtained from cases in the Gene Expression Omnibus (GEO) database (GSE14210 and GSE15457).

### 2.2 Bioinformatics analysis

RAB31 expression was assessed in different stages of disease using clinical data derived from GEPIA (http://gepia.cancer‐pku.cn/). RAB31 pathway‐related genes associated with GC tumor invasivity and metastasis were additionally explored through a gene set enrichment analysis (GSEA).

### 2.3 Cell culture

GC cells used for this study were obtained from the American Type Culture Collection. Cells were cultured in RPMI‐1640 (Gibco) containing 10% FBS in a 37°C 5% CO_2_ incubator.

### 2.4 Animals and experimental design

BALB/c mice were purchased from the Shanghai Model Organisms Center, Inc. For determination of whether RAB31 promoted metastasis of GC in mice, mice were randomly divided into two groups and treated as follows: (1) control, mice were injected with saline; (2) RAB31 OE, mice were injected with MGC803 cells (PRID: CVCL_5334, established from a 53‐year‐old male patient with poorly differentiated mucoid adenocarcinoma of the stomach) via the tail vein. For exploration of overexpression of RAB31 enhanced metastasis of GC by promoting exosome secretion, mice were randomly divided into four groups and treated as follows: (1) control, mice were injected with saline; (2) RAB31 OE, mice were injected with MGC803 cells via the tail vein; (3) NC‐Exo, mice were injected with exosomes secreted by the control group; (4) OE‐Exo, mice were injected with exosomes secreted by RAB31 overexpressing cells. Tissues of mice were collected and then stored at −80°C until use. UR52101 (Umibio, http://www.umibio.cn/productinfo/1575102.html) was used for preparing exosome samples. All experiments were approved by the laboratory animal ethical committee of Shanghai Sixth People's Hospital and followed the NIH Guide for Laboratory Animals for the Raise and Use of Mice.

### 2.5 Transfection

Cells were added to plates (1 × 10^5^/well) and incubated for 24 h, after which media was exchanged for serum‐free media, and a RAB31‐specific siRNA (Genepharm Technologies) were transfected into these cells using Lipofectamine 3000 (ThermoFisher) based on provided directions. At 6 h post‐transfection, media was exchanged for media supplemented with 10% FBS. Downstream assays were performed at 48 h post‐transfection to the manufacturer's instructions. For all siRNA and plasmid sequences used in this study, see Supplementary Material S1.

### 2.6 Transwell assays

After transfection, cells were added into Transwell inserts (1 × 10^5^/mL), with these inserts having been pre‐coated with 60 μL of fresh Matrigel (BD Biosciences) and incubated for 2 h in a 37°C tissue culture incubator for invasion assays. Cells in the upper chamber were suspended in a 200 μL volume, whereas the lower chamber was filled with 600 μL of media supplemented with 20% FBS. Following a 48 h incubation period, cells that had infiltrated the lower chamber were fixed for 15 min using 4% formaldehyde after using a cotton swab to eliminate cells in the upper chamber, after which 0.1% crystal violet was used to stain cells. Cells were subsequently imaged via microscopy, with average cell numbers in three random fields of view being recorded.

### 2.7 Western immunoblotting

RIPA buffer was used to extract total cellular protein, after which a BCA protein Assay Kit (ThermoFisher) was used to quantify protein concentrations, and samples were combined with protein loading buffer (WeiAo Company). Proteins were subsequently separated via 10% SDS‐PAGE, transferred to nitrocellulose membranes, and these blots were blocked for 2 h using 5% non‐fat milk at room temperature. Following overnight incubation with primary antibodies at 4°C, blots were probed for 1 h using secondary antibodies (WeiAo Company) at room temperature, after which protein bands were visualized with a chemiluminescent substrate (ThermoFisher).

This study utilized primary antibodies specific for RAB31 (16182‐1‐AP, Proteintech), CD63 (1:1000, ab134045, Abcam), CD81 (1:1000, ab79559, Abcam), TSG101 (1:1000, ab125011, Abcam), GAPDH (1:1000, ab8245, Abcam), PSMA1 (1:1000, ab109500, Abcam), N‐cadherin (1:1000, ab76011, Abcam), E‐cadherin (1:1000, 3195, CST), Snail (1:1000, 3879, CST), and vimentin (1:1000, 5741, CST).

### 2.8 qPCR

TRIzol (TaKaRa) was used to lyse tissues, after which chloroform and isopropyl alcohol were used for RNA extraction.

A NanoDrop 2000 (Thermo Scientific) was used to quantify RNA concentrations, after which reagents from TaKaRa were used to synthesize cDNA based on provided directions. SYBR Green and other reagents (TaKaRa) were then used to perform qPCR analyses using the RAB31‐specific primers listed in Supplementary Material S1.

### 2.9 Immunohistochemical (IHC) staining

Xylene and alcohol were used to deparaffinize the prepared tissue microarray, which was then rinsed for 15 min with water. Sections were microwaved with an appropriate buffer for antigen retrieval, treated for 15 min with an endogenous peroxidase blocker, and incubated overnight with primary anti‐RAB31 (1:100, 16182‐1‐AP, Proteintech) and anti‐PSMA1 (1:100, ab109500, Abcam) at 4°C followed by incubation for 1 h with an appropriate secondary antibody at room temperature. DAB and hematoxylin were then used to stain tissues, which were subsequently dried and imaged under a microscope. Two pathologists independently graded RAB31 IHC staining in a semi‐quantitative manner based on staining intensity (0, no staining; 1, weak; 2, moderate; 3, strong) and the percentage of RAB31‐positive tumor cells (0, none; 1, 1%–29%; 2, 30%–69%; 3, >70%). These scores were used to establish RAB31 expression as being either high (score: 4–9) or low (score: 0–3).

### 2.10 Nanoparticle tracking and electron microscopy analyses

Exosome‐containing samples were diluted using PBS, after which a ZetaView PMX 110 (Particle Metrix) instrument and the ZetaView 8.04.02 software were used to conduct a nanoparticle tracking analysis (NTA). The instrument was calibrated with 110 nm polystyrene particles, and these NTA measurements were assessed at 11 locations. The temperature was maintained at approximately 23°C and 37°C during these NTA measurements.

For electron microscopy analyses, exosomes were suspended in 2% PFA and added onto a copper mesh of Formvar carbon. The copper mesh was rinsed with PBS, stained, and imaged with an electron microscope at 80 kV.^6^

### 2.11 Mass spectrometry analyses

Samples were lysed using an appropriate lysis buffer with shaking and agitation for 400 s that was repeated three times. A BCA assay was then used to measure protein levels in these samples, after which 100 μg of protein for each sample was transferred into a new Eppendorf tube and 8 M urea was used to adjust the sample volume to 100 μL. Next, 2 μL of 0.5 MTCEP was added to each tube followed by a 1 h incubation at 37°C. Then, 4 μL of 1 M iodoacetamide was added per tube followed by a 40 min room temperature incubation while protected from light. Five volumes of chilled (−20°C) acetone were then added per sample, and proteins were allowed to precipitate at −20°C overnight, after which they were washed two times using cooled 90% aqueous acetone. Precipitates were then dissolved in 100 μL of 100 mM TEAB. Sequence‐level modified trypsin (Promega) was added at a 1:50 (enzyme:protein, w/w) ratio, and proteins were allowed to digest at 37°C overnight. The resultant peptide‐containing solution was desalted using C18 ZipTip, after which a Pierce™ quantitative colorimetric peptide assay (23275) was used for quantification. A SpeedVac was then used to lyophilize samples. Peaks Studio was used for label‐free quantification with a 1% false discovery rate (FDR). Relative peptide feature abundance (precursor peak area) was analyzed in multiple samples. Feature detection was individually performed individually for each sample by utilizing a high‐performance retention time comparison algorithm to ensure that features of the same peptides were compared across different samples.

### 2.12 Histological staining

Xylene, anhydrous ethanol, and 75% ethanol were used to deparaffinize tissue sections which were then rinsed for 15 min under tap water followed by staining with hematoxylin for 3–5 min. After a second rinse under tap water, sections were stained with differentiation solution, rinsed under tap water, turned blue with the appropriate solution, rinsed again, dehydrated for 5 min with an alcohol gradient (85% and 95%), and stained for 5 min with eosin. Anhydrous ethanol and xylene were then used to dehydrate sections which were subsequently sealed with neutral gum and imaged with a Nikon Eclipse E100 microscope.

### 2.13 RAB31‐overexpressing cell generation

A RAB31 overexpression plasmid was prepared by generating a primer pair to amplify the RAB31 coding sequence and cloning it into the lentiviral pCDH‐CMV‐MCS‐EF1‐GFP+Puro vector. The resultant RAB31 overexpression vector or an empty control vector was then used to transfect MGC803 cells, with a fluorescence microscopy subsequently being used to gauge transfection efficiency. Cell selection was then performed by incubating cells for 48 h in 5 μg/mL puromycin, followed by the transfer of selected clones to 96‐well plates for subsequent screening. Overexpression was validated via qPCR and Western immunoblotting, and the resultant data are provided in Supplementary Materials S1.

### 2.14 Statistical analysis

Data are means ± standard deviation (SD) and were compared with SPSS and GraphPad Prism. GC patient survival was analyzed using Kaplan–Meier curves, while independent risk factors were identified through Cox regression analyses. *p* < 0.05 was the cutoff threshold for statistical significance (Table 1).

**TABLE 1:** Correlation of RAB31 expression with the clinicopathological characteristics of gastric cancer.

RAB31 expression				
Characteristic	Category	Low	High	*p*‐value
		(*n* = 32%)	(*n* = 52%)	
Age (years)	≤50	4	7	0.99
	>50	28	45	
Sex	Male	21	40	0.316
	Female	11	12	
TNM	I/II	14	10	*0.024*
	III/IV	18	42	
Tumor location	Cardia	5	8	0.99
	Body	6	10	
	Antrum	17	28	
	Other site	4	6	
Lymph node metastasis	No	19	10	*<0.001*
	Yes	13	42	
Tumor size	≥5 cm	19	40	0.087
	<5 cm	13	12	
T stage	T1/T2	12	7	*0.011*
	T3/T4	20	45	
Distant metastasis	No	28	43	0.554
	Yes	4	9	
Lymphatic infiltration	No	27	28	*0.004*
	Yes	5	24	

*Note*: Italicized values indicate statistical significance when *p* < 0.05.

## 3 RESULTS

### 3.1 RAB31 overexpression is associated with more advanced GC and poorer prognostic outcomes

**FIGURE 1:**
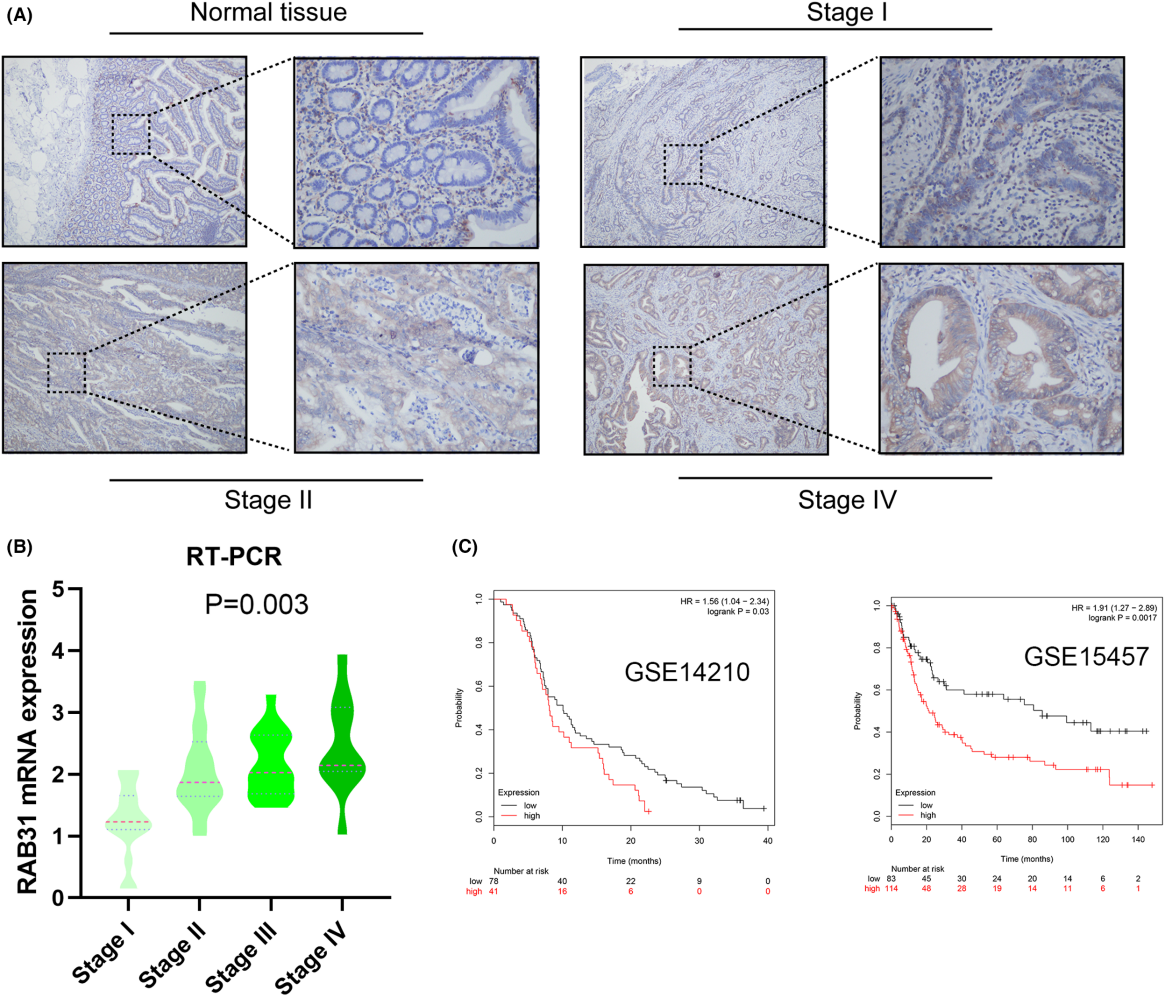
Overexpression of RAB31 predicted poorer prognosis in GC patients. (A) Representative images of immunohistochemistry staining of RAB31 expression normal and GC tissues in different stages of GC. (B) RT‐qPCR analysis of RAB31 mRNA expression levels in GC and para‐carcinoma tissues of 22 patients (*p* < 0.01). (C) Overall survival analysis of GC patients with low versus high RAB31 expression. Survival rate was calculated by Kaplan–Meier survival analysis (*p* < 0.001, log‐rank test). GC, gastric cancer; RT‐PCR, reverse transcription‐polymerase chain reaction.

Initial analyses of the GEPIA database revealed significant increases in RAB31 expression levels with GC progression.^2^ Consistently, qPCR analyses of 41 paired GC tumor and paracancerous tissues confirmed that the highest and lowest RAB31 expression levels were, respectively, evident in Stage IV and Stage I disease (Figure 1B). IHC staining similarly revealed that RAB31 expression levels were positively associated with GC progression (Figure 1A). These IHC results were used to separate patients into those expressing low and high levels of RAB31, revealing an association between the expression of RAB31 and lymph node metastasis (*p* = 0.0002), T stage (*p* = 0.01), and lymphatic vessel infiltration (*p* = 0.004). Elevated levels of RAB31 expression were also predictive of more advanced tumor staging, and Kaplan–Meier survival analyses confirmed that high levels of RAB31 expression were associated with poorer survival outcomes in both the present sample cohort (Figure S1; Table 2) and in two independent GEO datasets (GSE14210 and GSE15457) (Figure 1C).

**TABLE 2:** Univariate and multivariate analysis of prognostic factors for survival among patients with gastric cancer.

	Univariable analysis		Multivariable analysis	
	HR (95% CI)	*p*	HR (95% CI)	*p*
Age (years) (≥50 vs. <50)	1.025 (0.93–1.504)	0.487		
Sex (male vs. female)	1.101 (0.764–1.901)	0.454		
Tumor location (antrum vs. other parts)	0.707 (0.302–0.903)	0.056		
Tumor size (≥5 cm vs. <5 cm)	1.801 (1.511–3.037)	*0.039*	1.051 (0.766–1.28)	0.346
Distant metastasis (yes or no)	2.554 (1.351–4.661)	*0.008*	1.408 (0.975–2.049)	*0.044*
Lymph node metastasis (yes or no)	3.437 (1.811–4.671)	*0.000*	2.361 (1.207–3.407)	*0.000*
Lymphatic infiltration (yes or no)	2.737 (1.93–4.451)	*0.002*	1.781 (1.431–2.251)	*0.01*
TNM stage (I/II vs. III/IV)	2.145 (1.109–4.011)	*0.015*	1.536 (0.913–2.204)	*0.041*
T stage (T1/T2 vs. T3/T4)	1.86 (1.031–3.341)	*0.031*	0.897 (0.685–1.217)	0.391
Invasive depth (serosa vs. mucous/muscular layer)	1.945 (1.129–3.041)	*0.007*	1.645 (1.209–2.941)	*0.024*
RAB31	1.953 (1.382–3.542)	*0.034*	1.209 (0.567–2.057)	0.056
PSMA1 (high vs. low)	2.165 (1.124–5.038)	*0.004*	2.07 (1.591–3.346)	*0.01*

*Note*: Italicized values indicate statistical significance when *p* < 0.05.

Abbreviations: CI, confidence interval; HR, hazard ratio.

### 3.2 RAB31 expression levels are related to the migratory and invasive activity of GC cells

**FIGURE 2:**
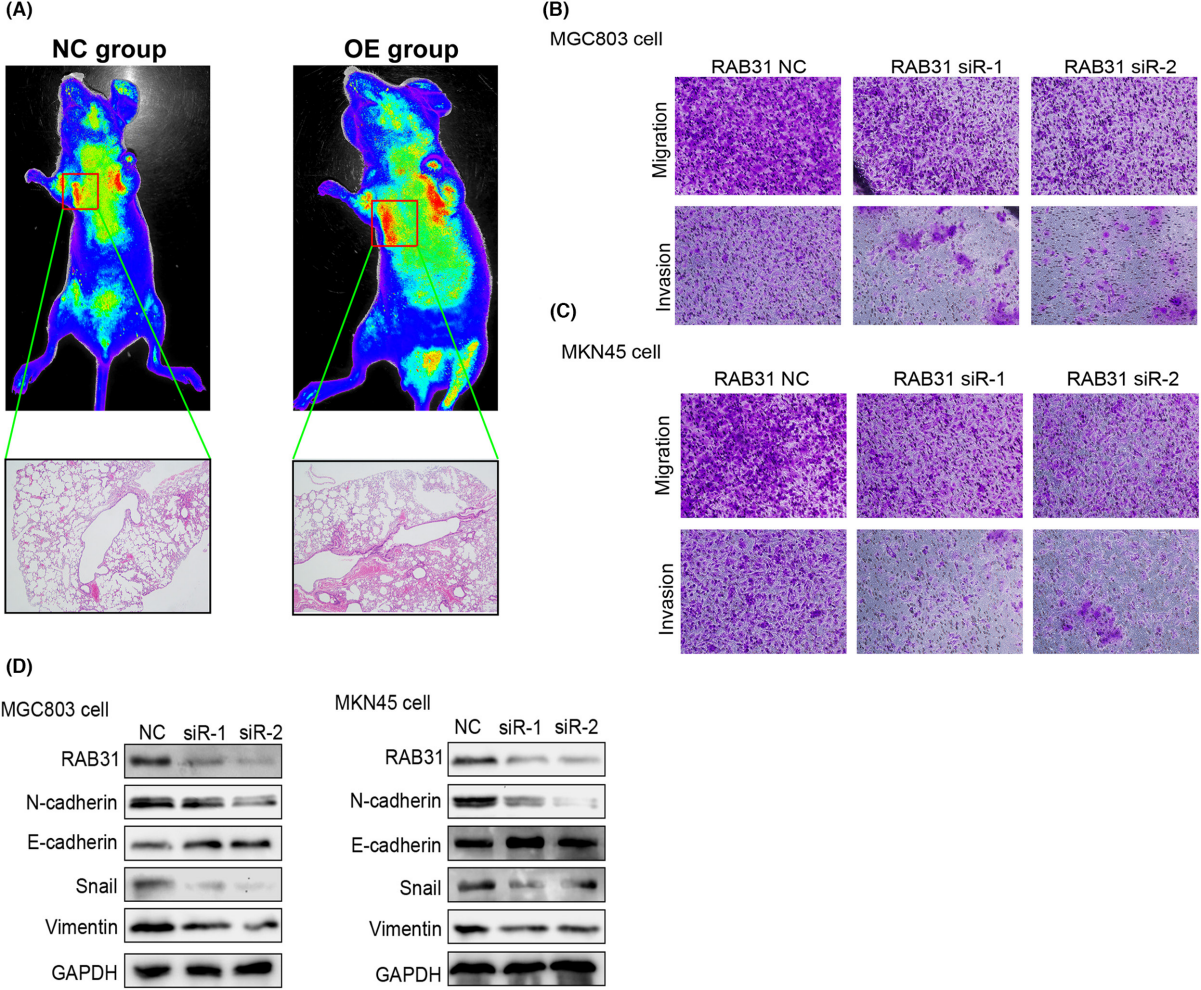
Overexpression of RAB31 promoted metastasis of GC in vivo and in vitro. (A) The pulmonary metastasis model of gastric cancer was established by injecting GC cells into nude mice via the tail vein. Representative images of HE staining of the NC and RAB31 OE groups. (B) Representative images showing the invasion and migration of MGC803 cells after knockdown of RAB31 expression. (C) Representative images showing the invasion and migration of MKN45 cells after knockdown of RAB31 expression. (D) Western blot analysis of EMT‐related proteins after knockdown of RAB31 expression.

Western immunoblotting and qPCR were used to confirm the successful establishment of cells stably overexpressing RAB31 (Supplementary Materials S1). These cells were then used to establish a murine model of pulmonary metastasis, and subsequent experiments revealed that RAB31‐overexpressing tumor cells gave rise to more pulmonary metastases and that these lesions were larger on average than those produced by control GC cells (Figure 2A). In line with these findings, MGC803 and MKN45 cell migratory and invasive activity was suppressed when RAB31 was knocked down (Figure 2B,C). Western immunoblotting further confirmed that knocking down RAB31 inhibited the expression of Vimentin, Snail, and N‐cadherin (Figure 2D).

### 3.3 RAB31 controls GC cell migration and invasivity by regulating the secretion of exosomes

**FIGURE 3:**
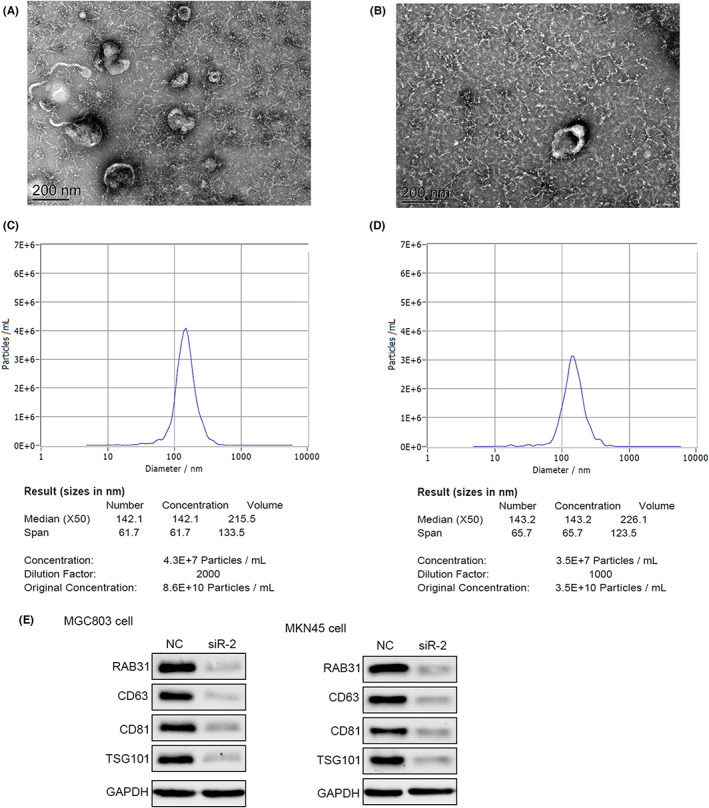
Depletion of RAB31 reduced the release of exosomes. (A and B) Representative electron microscopic images of exosomes in the normal control (NC) group (A), and the RAB31 depletion group (B). (C and D) Exosome nanoparticle tracking analysis of the NC group (C) and the RAB31 depletion group (D). Western blot analysis of exosome‐related proteins in the MGC803 cells and MKN45 cells between NC group and RAB31‐depleted group (E).

**FIGURE 4:**
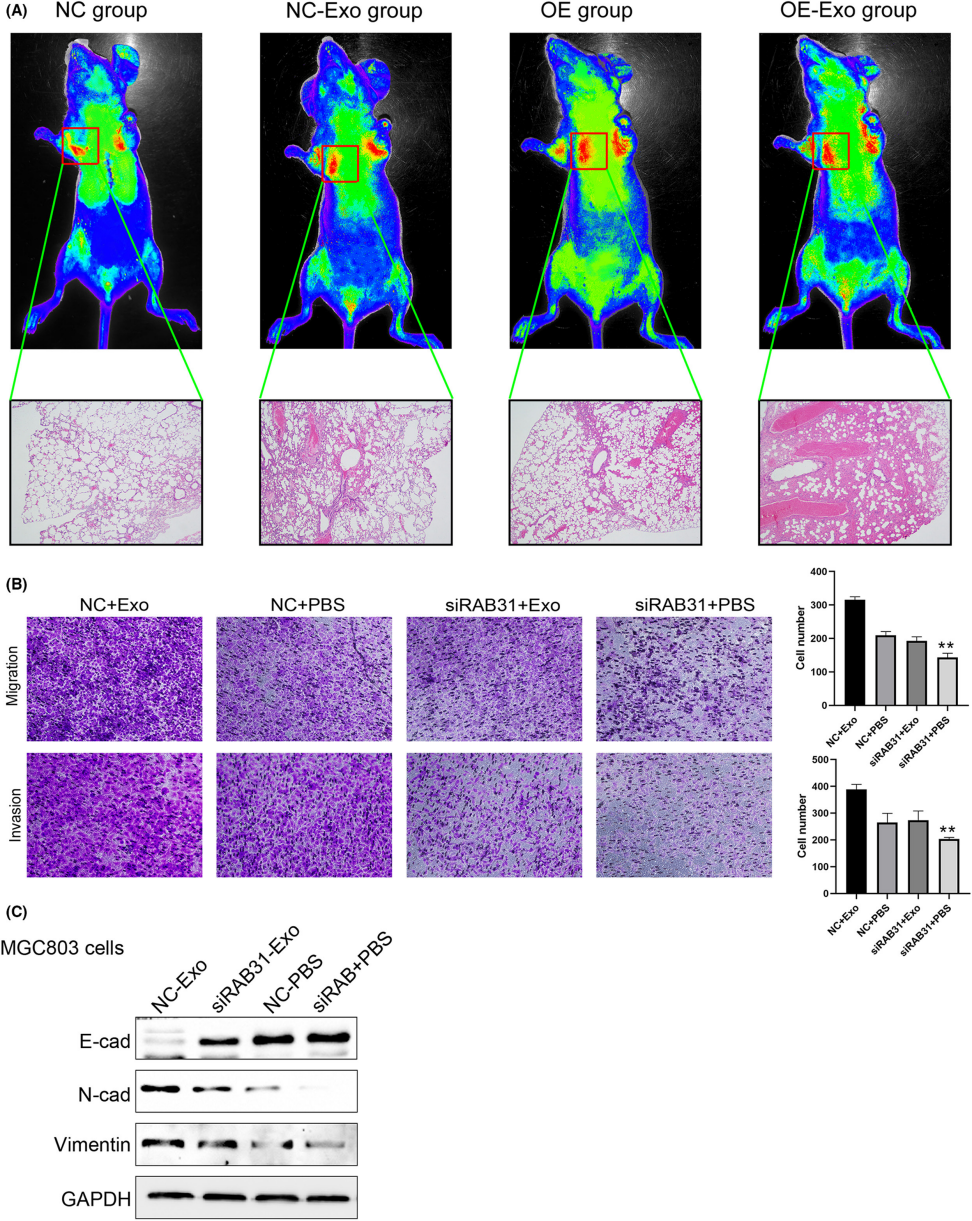
Overexpression of RAB31 enhanced metastasis of GC by promoting exosome secretion. (A) Pulmonary metastasis model of gastric cancer was established by injecting gastric cancer cells into nude mice via the tail vein. Representative images of HE staining of the NC, RAB31 OE, NC‐Exo (exosomes secreted by the control group), and OE‐Exo (exosomes secreted by RAB31 overexpressing cells) 20 groups. (B) Representative images of MGC803 and MKN45 cells in invasion and migration assays (left); data represent the mean ± standard deviation of three experiments (right) (*p* < 0.01). (C) Western blot analysis of EMT‐related proteins in the four groups of MGC803 cells described in (B).

RAB31‐knockdown cells were next utilized to assess the regulatory role of RAB31 in the context of exosome secretion through nanoparticle tracking analyses and electron microscopy. Following RAB31 knockdown, a drop in the size and number of exosomes released from these cells was observed (Figure 3A–D), with a concomitant decrease in the expression of certain exosomal marker proteins (Figure 3E). In our in vivo murine pulmonary metastasis model system, lung metastasis was enhanced by the injection of either RAB31‐overexpressing cells or supplementary exosomes as compared to that observed in mice injected with control MGC803 cells (Figure 4A). H&E staining revealed that the highest metastatic lesion burden was evident in mice injected with both RAB31‐overexpressing cells and exogenous exosomes, followed by mice injected with either of the two (Figure 4A). In vitro, MGC803 and MKN45 cell migration and invasivity were suppressed by the knockdown of RAB31, whereas exogenous exosome treatment was sufficient to reverse these changes (Figure 4B). Western immunoblotting additionally demonstrated that these functional phenotypes were also related to changes in the expression of the EMT marker proteins Vimentin and N‐cadherin (Figure 4C).

### 3.4 RAB31 expression is related to exosome protein cargo composition

**FIGURE 5:**
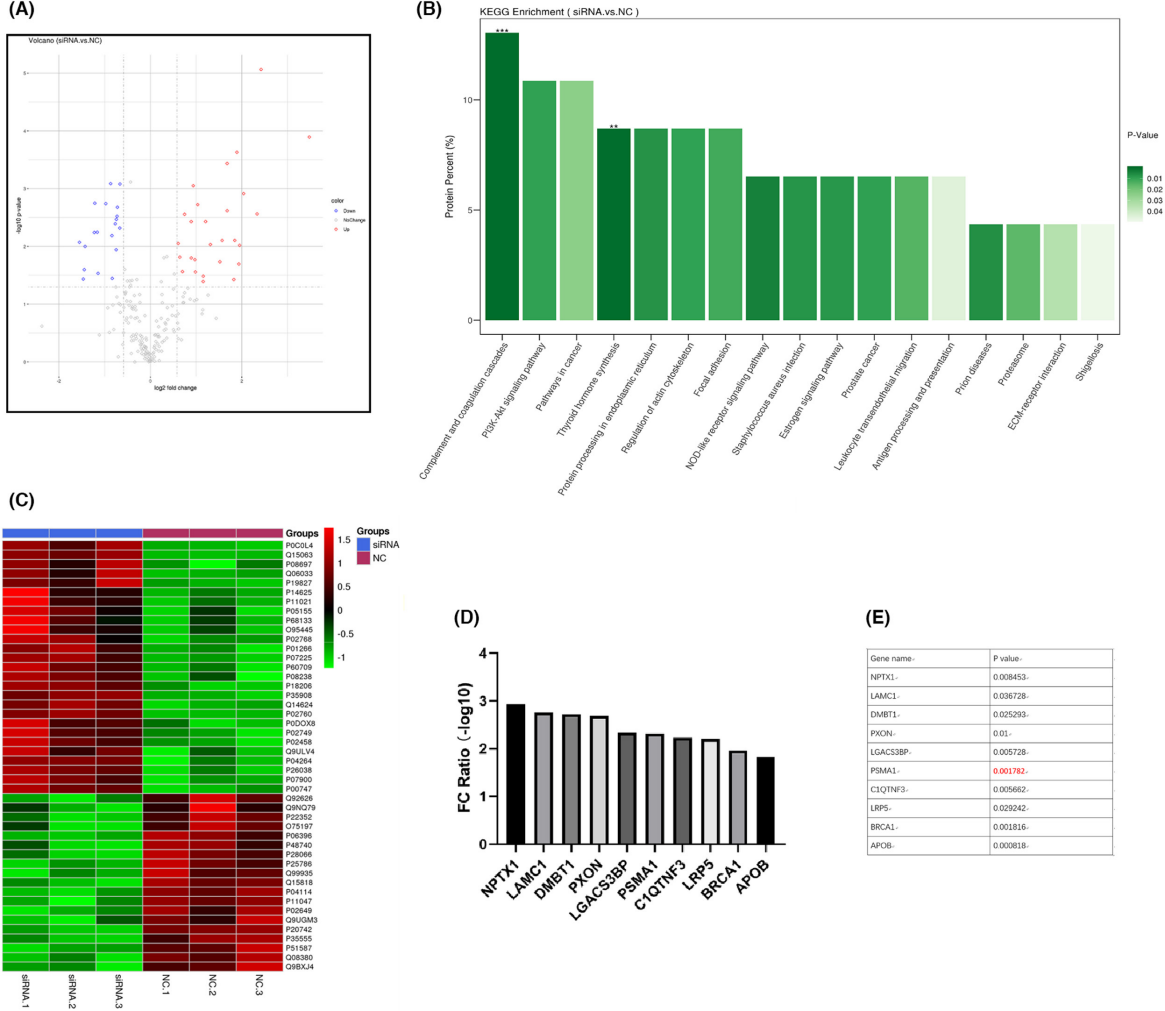
Protein mass spectrometry to identify the differentially expressed exosomal proteins between control group and RAB31 depleted group. (A) Volcano plot showing the differentially expressed proteins. (B) Histogram showing the results of the KEGG analysis. (C) Heat map showing the differentially expressed proteins. (D and E) Ranking of the differentially expressed proteins by FC value (D) and *p*‐value (E).

**FIGURE 6:**
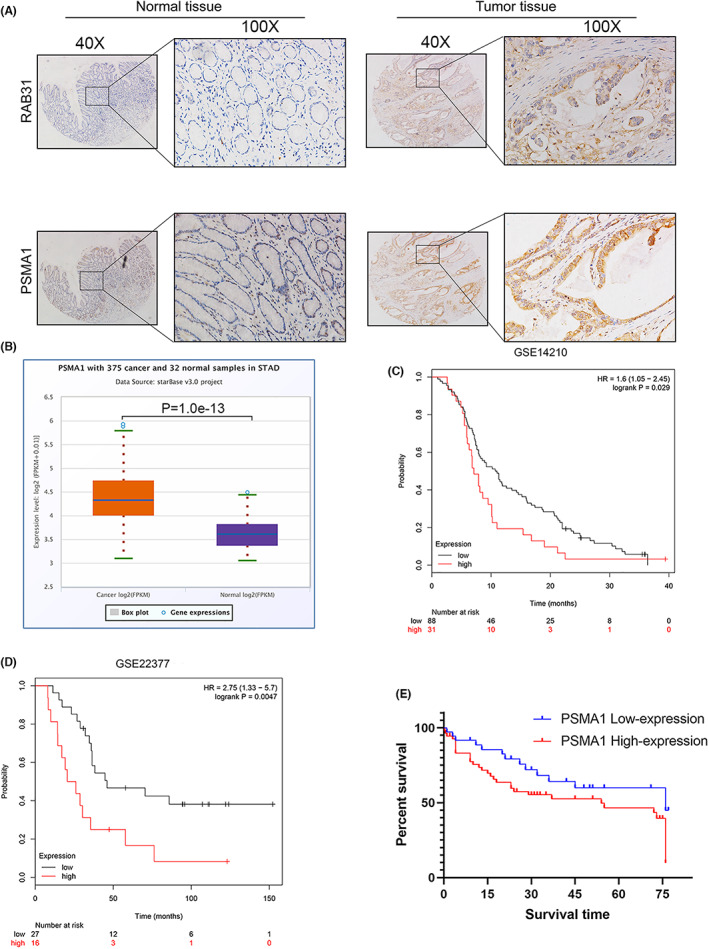
RAB31 expression was associated with PSMA1, and PSMA1 overexpression was associated with a worse prognosis. (A) Representative images of IHC staining of RAB31 and PSMA1 in gastric tumor and non‐tumor tissues. (B) PSMA1 mRNA level was assessed by TCGA database. (C and D) Overall survival analysis of GC patients with low versus high PSMA1 expression. Survival rate was calculated by Kaplan–Meier survival analysis (*p* < 0.001, log‐rank test). (E) Overall survival analysis of our GC patients with low versus high PSMA1 expression.

Mass spectrometry was next used to evaluate changes in exosomal protein cargo content when comparing samples harvested from RAB31‐knockdown and control cells. Specific proteins were downregulated and upregulated following the silencing of RAB31 (Figure 5A,C), and GO enrichment analyses suggested that a subset of these differentially abundant proteins was associated with the proteasome (Figure 5B). The proteasome‐related protein PSMA1 was highly correlated with the expression of RAB31 in these analyses (FC ratio >2, *p* < 0.01) (Figure 5D,E, Figure S2). PSMA1 IHC staining results from 84 paired GC and paracancerous tissues were used to stratify patients into those expressing low and high levels of PSMA1. Through this approach, PSMA1 expression levels were found to be strongly associated with TNM stage (*p* = 0.037), T stage (*p* = 0.002), and lymphatic vessel infiltration (*p* = 0.002) (Table 3). RAB31 levels were also positively correlated with the expression of PSMA1 in these GC tumor tissues (Figure 6A). In line with these findings, PSMA1 expression at the mRNA level was significantly increased in 375 GC tumor tissue samples relative to 32 control samples. Survival analyses performed using the GSE14210 and GSE22377 datasets additionally confirmed that the expression of high PSMA1 levels was associated with poorer prognostic outcomes as compared to the expression of low levels of this proteasome‐related protein (Figure 6C–E).

**TABLE 3:** Correlation of PSMA1 expression with the clinicopathological characteristics of gastric cancer.

PSMA1 expression				
Characteristic	Category	Low	High	*p*‐value
		(*n* = 36,42.9%)	(*n* = 48,57.1%)	
Age (years)	≤50	5	6	0.942
	>50	31	42	
Sex	Male	26	35	0.799
	Female	10	13	
TNM	I/I	13	11	*0.037*
	III/IV	23	37	
Tumor location	Cardia	6	7	0.902
	Body	8	8	
	Antrum	18	27	
	Other site	4	6	
Lymph node metastasis	No	16	13	0.097
	Yes	20	35	
Tumor size	≥5 cm	24	35	0.103
	<5 cm	12	13	
T stage	T1/T2	12	7	*0.002*
	T3/T4	14	41	
Distant metastasis	No	32	39	0.535
	Yes	4	9	
Lymphatic infiltration	No	30	25	*0.002*
	Yes	6	23	

*Note*: Italicized values indicate statistical significance when *p* < 0.05.

## 4 DISCUSSION

The present results reveal that the overexpression of RAB31 can predict more advanced GC disease progression and poorer prognostic outcomes in affected patients, and that these RAB31 expression levels are also related to GC cell invasivity and migration. Mechanistically, we demonstrated that RAB31 can control the migration and invasivity of GC cells by influencing the secretion of exosomes and that there may be a relationship between RAB31 levels and shifts in PSMA1 protein content in the exosomes released from these tumor cells.

GC remains one of the most prevalent and deadliest cancers globally and the second most common malignancy in China, accounting for an estimated 679,000 and 498,000 diagnoses and deaths throughout the world each year, respectively.^1^, ^7^ GC tumors are often highly invasive and prone to metastatic progression, contributing to poor patient outcomes in many cases. RAB31 is a non‐transformed monomeric protein member of the RAB family and the Ras superfamily that harbors four highly conserved domains that facilitate GTP binding and hydrolysis. Since its initial description in melanoma cells over two decades ago,^8^ RAB31 has been established as a regulator of the transport of many key proteins including glucose transporter type 4, epidermal growth factor receptor, and mannose 6‐phosphate receptors.^3^, ^4^, ^9^ High RAB31 expression levels have been reported in many tumor types and are reportedly closely associated with tumor TNM staging and prognostic outcomes.^10^, ^11^, ^12^, ^13^ In a prior study, we observed the overexpression of RAB31 in GC tumor samples in the GEPIA database.^2^ Supporting and expanding on this finding, we herein demonstrated through qPCR analyses of 41 paired GC tumor and paracancerous tissue samples that RAB31 expression levels were highest and lowest in Stage IV and Stage I GC, respectively. IHC analyses further confirmed a positive association between RAB31 protein levels and GC progression. In a murine model system, the overexpression of RAB31 enhanced GC tumor cell pulmonary metastasis, while in vitro experiments confirmed the role of RAB31 as a regulator of GC cell migration, invasivity, and EMT induction through experiments using cells in which this protein was knocked down.

The multi‐step progression from physiologically normal cells to malignant tumor cells is complex, and the survival, proliferation, and metastatic growth of these transformed cells are shaped to a significant degree by signals from the local microenvironment.^14^, ^15^ RAB family proteins serve as essential regulators of membrane vesicle transport within cells. For example, inhibiting the RAB27a/b proteins can alter the composition of the CD63+ compartment within cells and significantly suppress exosomal release.^16^ The TBCIDl0A‐C target protein RAB35 can similarly activate TBCIDl0A‐C exosome secretion.^17^ Accordingly, inhibiting RAB35 activity results in the intracellular accumulation of vesicles and the impairment of secretory processes.^18^ Therefore, we suppose that RAB31 maybe also have a function of regulating exosome secretion. Exosomes facilitate the exchange of potentially oncogenic macromolecules between tumor cells and their surrounding microenvironment. For example, in hepatocellular carcinoma exosomes have been documented as mediators of cell–cell communication and metastatic progression through their regulation of Wnt/β‐catenin signaling,^19^ while in esophageal cancer they regulate MAPK signaling,^20^ and they have been linked to poor GC patient prognostic outcomes.^5^ Here, RAB31‐knockdown cells were found to secrete fewer exosomes that were smaller in size on average. The injection of RAB31‐overexpressing GC cells or GC cell‐derived exosomes in vivo also enhanced tumor metastatic growth. In vitro, GC cell migration, invasivity, and EMT‐related protein expression were reduced following RAB31 knockdown, while exogenous GC cell‐derived exosome addition reversed these effects. These results thus support a role for RAB31 as a regulator of GC cell migratory and invasive activity through its ability to control exosome secretion.

Appropriate intracellular communication is highly dependent on the appropriate regulation of exosomal secretion,^21^ which is facilitated primarily through MVB biogenesis and trafficking. RAB family proteins including RAB11, RAB27, and RAB35 govern MVB transport to and docking with the lipid membrane, thereby regulating exosome release.^16^, ^17^, ^18^, ^22^ Here, a mass spectrometry approach was used to examine shifts in exosomal protein content in collected samples, revealing that TAB31 expression was strongly associated with the levels of the proteasome‐associated protein PSMA1 (FC ratio >2 and *p* < 0.01). IHC analyses further confirmed a relationship between PSMA1 expression and TNM stage (*p* = 0.037), T stage (*p* = 0.002), and lymphatic vessel infiltration (*p* = 0.002). Moreover, a positive correlation between RAB31 and PSMA1 expression levels was observed in GC tissues, and overexpressing PSMA1 was linked to a worse patient prognosis. In line with prior evidence, we also observed PSMA1 overexpression in exosomes collected from the serum of metastatic GC patients.^23^

In summary, these findings reveal novel biological mechanisms through which RAB31 can promote exosome release from GC cells, thereby enhancing the malignancy of GC cells through the enhancement of their invasive and metastatic potential. At the molecular level, the RAB31‐mediated regulation of exosomal PSMA1 content was identified as a candidate mediator of these observed phenotypes. Overall the results from this study offer clear evidence in support of the role of RAB31 as a coordinator of GC metastasis, implicating it as a promising prognostic biomarker and potential target for future therapeutic interventions aimed at improving GC patient outcomes.

## AUTHOR CONTRIBUTIONS

**Shan Wu:** Conceptualization (equal); formal analysis (equal); funding acquisition (lead); project administration (lead). **Chao‐Tao Tang:** Conceptualization (equal); validation (equal); writing – original draft (lead); writing – review and editing (lead). **Qian Zhuang:** Data curation (equal); formal analysis (equal); investigation (equal); software (equal); visualization (equal). **Qing‐Wei Zhang:** Conceptualization (equal); data curation (equal); resources (equal); software (equal). **Xin Ye:** Project administration (equal); software (equal); writing – original draft (equal). **Jie Xia:** Conceptualization (equal); data curation (equal); resources (equal); writing – original draft (equal). **Yan Shi:** Conceptualization (equal); data curation (equal); investigation (equal); resources (equal); writing – review and editing (equal). **Min Ning:** Data curation (equal); resources (equal). **Zhi‐xia Dong:** Conceptualization (equal); data curation (equal); resources (equal); writing – review and editing (equal). **Xin‐jian Wan:** Conceptualization (lead); funding acquisition (lead); writing – review and editing (equal).

## CONFLICT OF INTEREST STATEMENT

None.

## Supporting information


Supplementary Materials S1:

## Data Availability

Data sharing is not applicable to this article as no new data were created or analyzed in this study.
