# Supplementary material for: Overexpression of RAB31 in gastric cancer is associated with released exosomes and increased tumor cell invasion and metastasis
Source: Cancer Med. 2023 May 24;12(12):13497–510. doi: 10.1002/cam4.6007 (PMC10315821; doi:10.1002/cam4.6007)
Supplement: Supplementary file 1 — Supplementary Materials S1: [file CAM4-12-13497-s001.docx]

Supplementary materials 1

1. The primer sequences of RAB31 were as follows: forward 5′-ATCTTTGGGCTGGGTTTG-3′and reverse 5′-ATGGGCTCATTAGTGGGTAG-3′.

2. The siRNA sequences of RAB31 were as follows:

| Gene name | Sense sequence (5’-3’) | Anti-sense sequence (5’-3’) |
| --- | --- | --- |
| RAB31 | GGAGCUCAAAGUGUGCCUUTT | AAGGCACACUUUGAGCUCCTT |
| RAB31 | GGAAUACGCUGAAUCCAUAGGUGCC | GGCACCUAUGGAUUCAGCGUAUUCC |

3. The plasmid of RAB31 overexpression were as follows:

atcaatgggcgtggatagcggtttgactcacggggatttccaagtctccaccccattgacgtcaatgggagtttgttttggcaccaaaatcaacgggactttccaaaatgtcgtaacaactccgccccattgacgcaaatgggcggtaggcgtgtacggtgggaggtttatataagcagagctcgtttagtgaaccgtcagatcgcctggagacgccatccacgctgttttgacctccatagaagattctagagctagcgaattcgccaccatgatggcgatacgggagctcaaagtgtgccttctcggggacactggggttgggaaatcaagcatcgtgtgtcgatttgtccaggatcactttgaccacaacatcagccctactattggggcatcttttatgaccaaaactgtgccttgtggaaatgaacttcacaagttcctcatctgggacactgctggtcaggaacggtttcattcattggctcccatgtactatcgaggctcagctgcagctgttatcgtgtatgatattaccaagcaggattcattttataccttgaagaaatgggtcaaggagctgaaagaacatggtccagaaaacattgtaatggccatcgctggaaacaagtgcgacctctcagatattagggaggttcccctgaaggatgctaaggaatacgctgaatccataggtgccatcgtggttgagacaagtgcaaaaaatgctattaatatcgaagagctctttcaaggaatcagccgccagatcccacccttggacccccatgaaaatggaaacaatggaacaatcaaagttgagaagccaaccatgcaagccagccgccggtgctgttgaggatccgcggccgcgaaggatctgcgatcgctccggtgcccgtcagtgggcagagcgcacatcgcccacagtccccgagaagttggggggaggggtcggcaattgaacgggtgcctagagaaggtggcgcggggtaaactgggaaagtgatgtcgtgtactggctccgcctttttcccgagggtgggggag

Supplementary materials 2


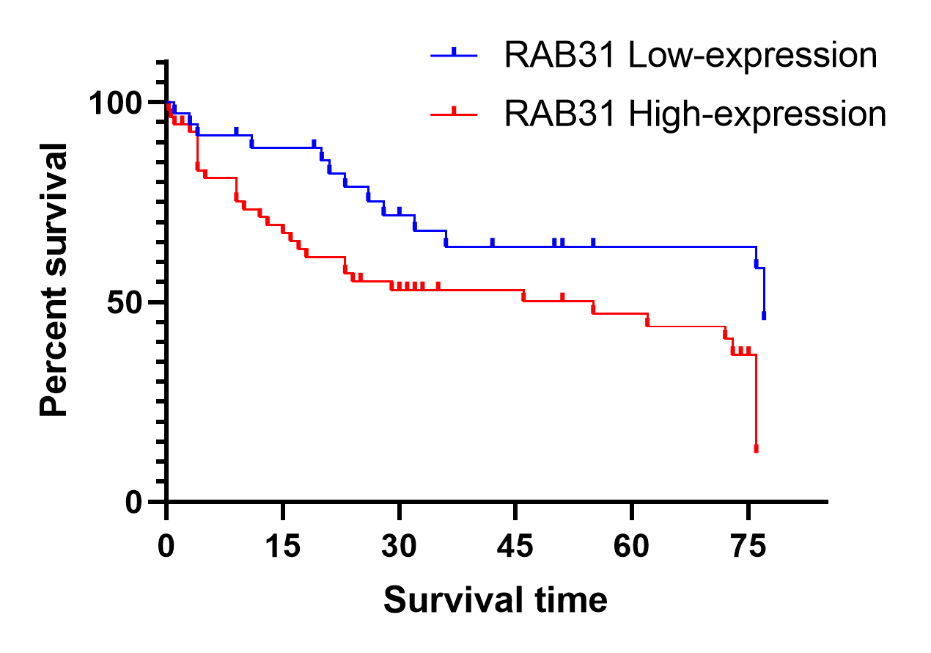


**Supplementary Figure 1:** Overall survival analysis of 84 GC patients with low versus high RAB31 expression. Survival rate was calculated by Kaplan–Meier survival analysis (P = 0.034, log-rank test).


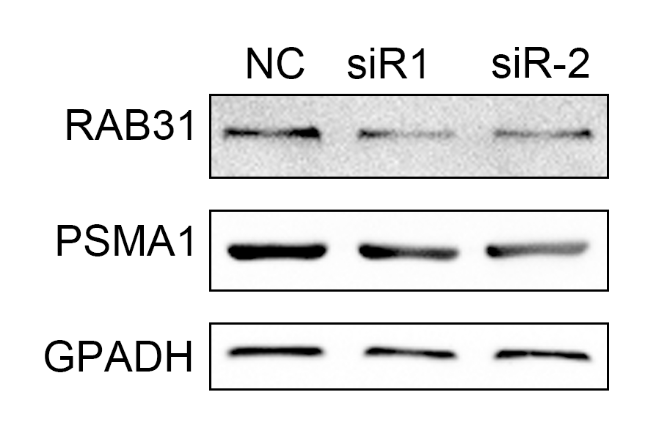


**Supplementary Figure 2:** Western blot analysis of correlation of RAB31 and PSMA 1.


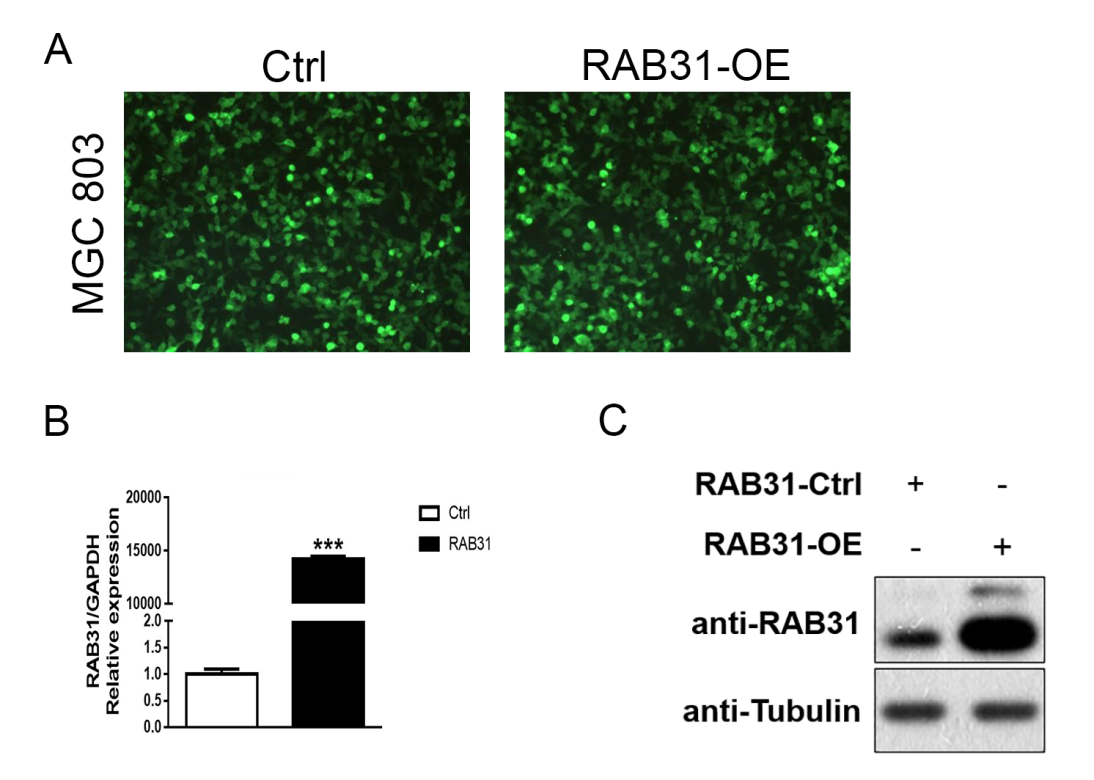


**Supplementary Figure 3: Constructing MGC803 cell model with stable expression of RAB31. A** Fluorescence microscope was used to detect the fluorescence intensity of MGC 803 cell after transfecting lentivirus. **B** RT-PCR assay was used to measure the expression of RAB31 mRNA between control and RAB31-overexpressed group. **C** Western blot assay was used to measure the expression of RAB31 protein between control and RAB31-overexpressed group
